# Multicolour Single Molecule Imaging in Cells with Near Infra-Red Dyes

**DOI:** 10.1371/journal.pone.0036265

**Published:** 2012-04-25

**Authors:** Christopher J. Tynan, David T. Clarke, Benjamin C. Coles, Daniel J. Rolfe, Marisa L. Martin-Fernandez, Stephen E. D. Webb

**Affiliations:** Central Laser Facility, Rutherford Appleton Laboratory, Science and Technology Facilities Council, Research Complex at Harwell, Didcot, United Kingdom; University of Milano-Bicocca, Italy

## Abstract

**Background:**

The autofluorescence background of biological samples impedes the detection of single molecules when imaging. The most common method of reducing the background is to use evanescent field excitation, which is incompatible with imaging beyond the surface of biological samples. An alternative would be to use probes that can be excited in the near infra-red region of the spectrum, where autofluorescence is low. Such probes could also increase the number of labels that can be imaged in multicolour single molecule microscopes. Despite being widely used in ensemble imaging, there is a currently a shortage of information available for selecting appropriate commercial near infra-red dyes for single molecule work. It is therefore important to characterise available near infra-red dyes relevant to multicolour single molecule imaging.

**Methodology/Principal Findings:**

A range of commercially available near infra-red dyes compatible with multi-colour imaging was screened to find the brightest and most photostable candidates. Image series of immobilised samples of the brightest dyes (Alexa 700, IRDye 700DX, Alexa 790 and IRDye 800CW) were analysed to obtain the mean intensity of single dye molecules, their photobleaching rates and long period blinking kinetics. Using the optimum dye pair, we have demonstrated for the first time widefield, multi-colour, near infra-red single molecule imaging using a supercontinuum light source in MCF-7 cells.

**Conclusions/Significance:**

We have demonstrated that near infra-red dyes can be used to avoid autofluorescence background in samples where restricting the illumination volume of visible light fails or is inappropriate. We have also shown that supercontinuum sources are suited to single molecule multicolour imaging throughout the 470–1000 nm range. Our measurements of near infra-red dye properties will enable others to select optimal dyes for single molecule imaging.

## Introduction

Single molecule imaging allows the behaviour of fluorescently labelled biomolecules to be studied without losing information through ensemble averaging. Molecular diffusion, interactions, the formation of complexes, molecular structure and conformational changes can all be studied through fluorescence microscopy at the single molecule level [1].

Because the fluorescence signal emitted by single organic dye molecules is so weak, a high signal to background ratio (SBR) also requires that the background signal is very low. In cells, a major contribution to the fluorescence background is from autofluorescence from within the cell. There are therefore two main strategies to maximising the SBR – a spatial approach, restricting excitation to the volume of interest, and a spectral approach, using excitation wavelengths that do not generate autofluorescence.

The most common technique for imaging single molecules in cells is Total Internal Reflection Fluorescence (TIRF) microscopy [2], in which an evanescent field is created which limits excitation to a thin layer a few hundred nanometres thick. This has been used to study proteins in both the apical and basolateral membranes of live cells [3], but TIRF is unable to follow these molecules as they internalise or to image organelles within the cell. Light sheet microscopy is an alternative method of limiting fluorescence excitation and has been used to image deeper planes [4], but requires complex setups.

The spectral approach involves exciting fluorescence in the near infra-red (NIR) region of the spectrum, where the background generated by cellular autofluorescence is much lower. This could be achieved either by two-photon excitation of visible fluorophores, as has recently been demonstrated with EGFP in fixed cells [5], or by using NIR probes.

NIR probes are routinely used in ensemble fluorescence imaging, particularly of thick samples, because absorption, autofluorescence and scattering are all lower in biological cells and tissue at longer wavelengths [6]. However, they have seen little use thus far in single molecule fluorescence studies. Using NIR organic dyes in single molecule imaging allows a high-fraction of molecules of interest to be extrinsically labelled without inducing toxic effects in cells and to exploit the advantages of their small size, which makes them less likely to interfere with inter-molecular interactions. Cy7 has been used in single molecule FRET studies [7]–[9], but since it was used only as an acceptor and in vitro, the spectral properties of cells were not relevant. Alexa 680, Cy7 and Alexa 750 have been used in stochastic optical reconstruction microscopy (STORM) of fixed cells and tissue, in which one step of image formation is the localisation of single molecules in acquired data [10], [11]. Choosing probes for single molecule fluorescence involves balancing their brightness, photostability and spectral characteristics to maximise the signal-to-noise ratio (SNR) - and NIR probes are no different. While these properties are often provided by manufacturers or can be found in the literature for visible fluorophores [12]–[16], there is little information to enable an optimal selection of NIR dyes for single molecule imaging. (Selected data for certain dyes specifically for the related technique of STORM were given in a recent study [17]). Furthermore, these properties may have been measured under different conditions than those used in a specific single molecule experiment. For instance, the quantum yield of Cy5 is variously reported as 0.18 [18], 0.28 [17] and 0.30 [19]. This variation may be due to the strong temperature dependence of Cy5's quantum yield [20].

Multiple fluorophores are often imaged simultaneously in multispectral microscopes so that the behaviours of different proteins may be correlated. Many of the studies noted above used a single NIR probe, together with visible ones, simply to image a larger number of bright fluorophores without spectral overlap than is possible within the visible part of the spectrum alone. However, it would often be desirable to perform multispectral single molecule imaging solely with NIR fluorophores, when background fluorescence is significant.

We have investigated the brightness, photostability and blinking characteristics of a range of commercially available, organic NIR dyes under the same conditions with a view to selecting the most appropriate for single molecule imaging in cells. We selected dyes that were compatible with multicolour NIR imaging, specifically dyes that could be excited at 695 nm or 780 nm. We demonstrate the use of the optimal dyes in each wavelength band for single molecule imaging in MCF-7 cells, a commonly used breast cancer cell line that produces a noticeable autofluorescence background when illuminated with visible wavelength light. In addition, we show that epi-illumination at NIR wavelengths is compatible with single molecule detection and discuss its advantages.

## Results

### Ensemble fluorescence properties of NIR dyes

The dyes selected for excitation at 695 nm were Alexa 700, Atto 700, DyLight 680 and IRDye 700DX; those excited at 780 nm were Alexa 790, CF790, DyLight 800 and IRDye 800CW. Their ensemble properties are summarised in Table 1. In both wavelength bands, the IRDye and Dylight fluorescence absorption and emission spectra are slightly blue-shifted from Alexa 700 and Atto 700, or Alexa 790 and CF 790 (Figure S1A).

**Table 1:** Ensemble fluorescence properties of NIR dyes.

Fluorescent dye	Absorption maximum/nm	Emission maximum/nm	ε_A_ at absorption maximum/M^−1^cm^−1^	Relative Quantum Efficiency	Brightness/(×10^5^) M^−1^cm^−1^
Alexa 700	693	719	192,000	0.37±0.04	0.70±0.08
Atto 700	699	720	120,000	0.21±0.03	0.25±0.03
DyLight 680	678	708	140,000	0.29±0.04	0.26±0.03
IRDye 700DX	689	702	165,000	0.44±0.06	0.61±0.08
Alexa 790	778	808	260,000	0.63±0.14	1.60±0.21
CF790	784	811	210,000	0.58±0.16	1.20±0.17
DyLight 800	771	798	270,000	0.34±0.09	0.80±0.11
IRDye 800CW	776	800	240,000	0.54±0.18	1.21±0.21

The optical properties of selected NIR dyes. Absorption and emission maxima were measured from samples of dyes dissolved in PBS. Molar absorption coefficients, ε_A_, at the peak absorption wavelength were obtained from the manufacturers. The brightness of dyes was estimated for excitation at 695 or 780 nm as appropriate.

When selecting dyes for single molecule fluorescence imaging, ensuring that an adequate signal from each molecule can be obtained is paramount. We estimated the relative brightness of the different dyes when excited at either 695 or 780 nm using ensemble solution measurements of their relative quantum yields. Plotting absorption vs. integrated fluorescence emission for a range of dye concentrations, the relative quantum yield is the gradient, normalised to that of a reference dye. Because it was not possible to calibrate the reference dye against a second standard, the quantum efficiencies given in Table 1 should be interpreted only as relative to each other within each group of four dyes at room temperature, rather than as absolute values. From this, the relative brightness of the dyes was determined at 695 or 780 nm (Figure S1B). Of the shorter wavelength band dyes, Alexa 700 and IRDye 700DX were found to be the brightest fluorophores at 695 nm, while Alexa 790 and IRDye 800CW were the brightest at 780 nm.

### Single molecule fluorescence properties of the Alexa 700, Alexa 790, IRDye 700DX and IRDye 800CW

The four brightest NIR dyes were selected for further analysis. Specifically, we investigated the single molecule intensity, photobleaching rate and blinking characteristics of immobilised dye molecules.

From images of single molecule features, histograms of feature intensities (after background subtraction) were generated (Figure S2) that displayed clear peaks. The histograms could be fit by summed Gaussian distributions, each corresponding to a different number of molecules within the feature [21]. The position of the first peak was interpreted as being the mean intensity of a single molecule. The mean intensities were converted to the mean number of photons detected per single molecule and the values for each dye are are reported in Table 2. Both IRDyes had mean feature intensities superior to the corresponding Alexa dye, although the difference between single Alexa 700 and IRDye 700DX molecules was minimal.

**Table 2:** Single molecule fluorescence properties of NIR dyes.

Fluorescent Dye	Mean number of photons per single molecule	Apparent photobleaching time constant/s	Mean emission period/s	Mean duty cycle
Alexa 700	57±0.5	2.2±0.1	0.5±0.1	0.62
IRDye 700DX	59.5±0.5	7.1±0.2	1.4±0.1	0.79
Alexa 790	82±0.5	4.3±0.1	1.6±0.1	0.87
IRDye 800CW	106±0.5	4.0±0.1	2.4±0.2	0.94

The measured single molecule fluorescence properties of the two brightest NIR dyes tested, in each wavelength band. The mean number of photons per single molecule was calculated from the mean single molecule fluorescence intensity integrated over 250 ms. The laser power exiting the objective was ∼1.6 µWµm^−2^ for all dyes. The mean number of photons per single molecule, the mean period of continuous fluorescence emission and the mean duty cycle were obtained from the intensity traces of 74 Alexa 700, 276 IRDye 700DX, 99 Alexa 790 and 272 IRDye 800CW molecules.

Next, we examined the photobleaching of the dyes. The total intensities of all detected features were plotted as a function of time for each dye (Figure S3). Two processes contribute to the form of these curves: irreversible photobleaching and long period blinking where molecules transition to a reversible dark state and back. A single exponential decay curve was fit to the data to obtain the apparent photobleaching time constants (the apparent average time before a molecule enters an irreversible dark state) in the presence of blinking (Table 2). Under TIRF illumination, we found that IRDye 700DX is more photostable than Alexa 700, whereas IRDye 800CW and Alexa 790 displayed similar photobleaching kinetics.

To investigate the contribution of blinking to the measured photobleaching kinetics, histograms of fluorescence emission periods were produced from the same datasets (Figure S4). In each case, a few molecules had emission periods equal to the total acquisition time. Features tracked for fewer than 5 frames were not included in this analysis. A single exponential decay curve was fit to these histograms to obtain the mean time that each dye continuously emitted before entering a dark state (the ‘on-time’). Additionally, the duty cycle for each molecule was defined as the summed emission periods divided by the time elapsed when the final bleaching event occurs. Both sets of results are shown in Table 2. Alexa 700 had an average ‘on-time’ of less than a second and a duty cycle of 0.62, i. e. Alexa 700 intensity traces comprise short bursts of fluorescence with dark periods lasting several seconds. In comparison, IRDye 700DX had an average ‘on-time’ of 1.4 seconds and a higher duty cycle, indicating that the dye emits for longer periods, punctuated by brief periods in a dark state. Of the longer wavelength dyes, we found that IRDye 800CW had a marginally longer ‘on-time’ and a greater duty cycle than Alexa 790. The duty cycle of IRDye 800CW was calculated to be close to one because a large number of molecules did not blink before irreversibly photobleaching

### Single molecule imaging with NIR dyes in cells

**Figure 1:**
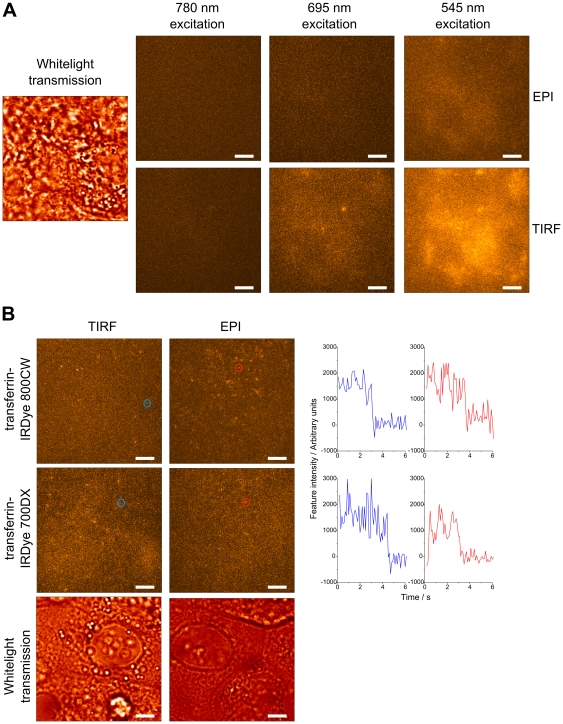
Autofluorescence background and single NIR molecules in MCF-7 cells. **A**. Images of a single area of unlabelled MCF-7 cells independently illuminated with broadband 780 nm, 695 nm and 545 nm light demonstrate a decrease in autofluorescence background with increasing excitation wavelength. A whitelight transmission image indicates the position of cells within the field of view. **B**. Images of single molecules of transferrin-IRDye 700DX and transferrin-IRDye 800CW on MCF-7 cells simultaneously illuminated with broadband 780 nm and 695 nm light. Typical intensity vs. time traces, of the molecules highlighted by red and blue circles, are shown to the right. Scale bars represent 2 µm.

We tested the assumption that there will be an improvement in the SBR in cell samples when using NIR dyes by imaging MCF-7 cells in the two NIR channels used previously, plus a third channel suitable for orange fluorescent dyes excited at 545 nm. Employing epi-illumination with each wavelength independently, we observed the expected decrease in the autofluorescence background from unlabelled cells with increasing wavelength. In the longest wavelength channel, there was almost no background above the noise of the detector when the cells were excited with 780 nm light (Figure 1A). This trend was also seen using TIRF illumination, but the autofluorescence signal is greater because the evanescent intensity is greater than the incident light intensity [2]. The existence of a significant fluorescence signal under TIRF illumination shows that, unlike some other cells, MCF-7 cells are strongly autofluorescent in superficial regions.

Next, MCF-7 cell membranes were labelled with 0.5 nM transferrin-IRDye 700DX and 0.5 nM transferrin IRDye 800CW (total concentration 1.0 nM). Transferrin is a glycoprotein that binds iron very tightly but reversibly and is taken into cells by receptor mediated endocytosis [22]. Transferrin was allowed to bind to its receptor at 4°C to inhibit receptor mediated endocytosis and the cells were subsequently fixed to trap transferrin receptors at the cell surface. Examples of single NIR molecules on cells observed with epi-fluorescence and TIRF illumination when simultaneously illuminated with 695 and 780 nm centred light are shown in Figure 1B. The background signal in the longer wavelength channel is greater than in figure 1A, which we attribute to bleedthrough of autofluorescence excited at 695 nm. However, the total background intensity still reduces with increasing wavelength with both epi and TIRF illumination. Single molecules of both dyes are clearly visible above background with TIRF illumination. Although it appears that IRDye 700DX may not be bright enough to be imaged with the lower intensity epi-fluorescence illumination, the IRDye 800CW maintains a sufficient SBR when imaging in this mode.

## Discussion

A wide range of near-infra-red fluorophores are commercially available for labelling proteins in biological cells and tissue. Because they have generally been designed for use in relatively high concentrations for ensemble fluorescence imaging, little is known of their photophysical characteristics which are relevant to their use in single molecule microscopy. In this study, we have studied example organic fluorophores suited to two-colour imaging with excitation at ∼695 nm and ∼780 nm. In order to provide a meaningful comparison, we used the same conditions for each fluorophore. We attribute the different trends regarding the IRDyes reported elsewhere [23] to the use of different conditions, such as temperature, which were not included. Other NIR fluorescent tags – quantum dots [24] and expressible proteins [25] – also exist, with the same advantages and disadvantages as their visible versions, but have not been considered here.

Because the signal-to-noise ratio (SNR) of the fluorescence from individual molecules is relatively poor, their brightness is critical to the success of a single molecule fluorescence experiments. Our determination of the quantum yields of each dye enabled us to select the most appropriate dyes for further characterisation relevant to single molecule microscopy. The blinking characteristics are particularly important when studying proteins free to diffuse in live cells. The tracks followed by these proteins are often highly irregular and cross each other frequently. If two tracks cross during blinking events, it is not generally possible to determine which tracks before and after the dark period should be paired. The photobleaching rate is also crucial – if the molecules bleach very fast, it is not possible to observe the behaviour of the protein that the dye is attached to for sufficiently long periods for diffusive properties to be derived. Alternatively, very short camera integration times are required, which reduces the SNR.

As Figure 1A shows, autofluorescence can be present in superficial parts of the cell as well as deeper compartments and hence restricting the illumination volume will not always assist in improving the SBR. With NIR fluorescent probes, however, it is possible to avoid exciting autofluorescence altogether, thus enabling single molecule imaging in samples with a high background intensity. We note that there is still a small background signal with 695 nm excitation, but this is negligible with 780 nm excitation. We believe that this is the first reported wide-field excitation of single molecules with wavelengths longer than 755 nm. Furthermore, it is no longer necessary to use a method of restricting the illumination volume in order to achieve single molecule sensitivity, although it may still be desirable to take advantage of the increased excitation intensity possible with TIRF illumination. In conjunction with methods for resolving the axial position of single molecules, which include astigmatism [26], multifocal imaging [27] and double-helix point spread functions [28], epi-illumination with NIR probes should enable 3D imaging of individual proteins in cells and tissue. We note that wide-field microscopes, as used here, have intrinsically faster data acquisition rates than scanning microscopes and hence are more appropriate for imaging dynamic samples.

Multicolour single molecule microscopes utilising a single NIR probe were referred to in the introduction. Our work to characterise other NIR dyes enables other combinations of fluorophores to be considered, although this is partly dependent on suitable filter sets being available. We note that the use of a supercontinuum white-light laser enables all fluorophores absorbant in the 470–1000 nm range to be optimally excited, i. e. at their excitation maxima, and so dye selection is independent of the laser wavelengths available. Furthermore, it is simpler to integrate a single supercontinuum laser into a microscope than it is to couple multiple single-wavelength lasers. This work extends the use of a supercontinuum source for imaging single molecules from the visible spectrum [29] to the NIR. It would be desirable to image more than two fluorophores within the NIR spectrum, but this has only been achieved by spectral unmixing in software [30], for which the SNRs required are not found in single molecule microscopy. If other NIR dyes are developed, with spectra further into the infra-red, the supercontinuum would be capable of exciting them too and the number of NIR fluorophores that could be imaged simultaneously would increase.

## Materials and Methods

### Near infra-red dyes and dye-protein conjugations

Alexa 700 and Alexa 790 NHS ester were obtained from Invitrogen. Atto 700 NHS ester was obtained from Atto-Tec. DyLight 680 and DyLight 800 NHS esters were obtained from Thermo Scientific. CF790 succinimidyl ester was obtained from Biotium. IRDye 700DX and IRDye 800CW were obtained from Li-Cor.

Alexa 700, Alexa 790, IRDye 700DX and IRDye 800CW were conjugated to biotin-transferrin using NHS-ester dye derivatives, following the manufacturer's instructions. Freed dye was separated from the conjugates using a PD-10 desalting column (GE Healthcare). The ratio of dye to protein for each conjugate was determined from its absorption spectra and was found to be 2∶1 for the transferrin-Alexa 700 and transferrin-IRDye 700DX conjugates and 1∶1 for the Alexa 790 and IRDye 800CW conjugates.

### Estimation of the relative brightness of dyes in solution

5 samples of each dye in PBS were prepared over a range of concentrations such that the peak absorbance of the highest concentration sample was <0.1 to avoid inner filter effects. Absorption spectra were recorded with a V-630 UV-Vis Spectrometer (Jasco). Emission spectra were recorded when samples were excited at either 625 nm or 725 nm with an AOTF-coupled supercontinuum light source (SC-450-8, Fianium), using a CCS-175 fibre spectrometer (Thorlabs) coupled to a cuvette holder (Ocean Optics). The integrated fluorescence emission for each sample was plotted as a function of its absorbance at the excitation wavelength. The excitation intensity was kept constant and the fluorescence from comparable dyes was corrected for the transmission profile of the spectrometer and integrated over a common wavelength range. The gradient of these plots was therefore proportional to the quantum yield of the dye. To check the consistency of the results, the gradients of Alexa 700, Atto 700, DyLight 680 and IRDye 700DX were compared to that of OX170 (Quantum yield, 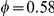 [31]). The gradients of Alexa 790, CF790, Dylight 800 and IRDye 800CW were compared to that of HITC Iodide (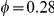 [31]). The two reference dyes were not cross referenced. The relative brightness, *B*, of each dye was calculated using 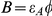, where 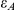 is the absorption coefficient at the excitation wavelength.

### Dye Immobilisation

35 mm glass bottomed dishes (MatTek) were cleaned with Piranha solution before being covered with 1 mg/ml BSA-biotin in Tris-EDTA (TE) buffer for 10 minutes at room temperature. After washing with TE buffer, dishes were covered with 0.2 mg/ml streptavidin in TE buffer for 10 minutes. Dishes were washed again in TE buffer before being incubated with a 0.05 nM solution of a single biotin-transferrin-NIR dye conjugate for 10 minutes at room temperature. Excess dye conjugate was washed off with TE buffer and immobilised dye samples were stored at 4°C before imaging.

### Cell sample preparation

MCF-7 cells ([32], European Collection of Cell Cultures) were cultured on 35 mm glass bottomed dishes (MatTek) in RPMI 1640 media supplemented with glutamine and fetal calf serum, at 37°C in the presence of 5% CO_2_. Cells were incubated in media without serum for 18 hours before being labelled with 0.05 nM biotin-transferrin-IRDye 700DX and 0.05 nM biotin-transferrin-IRDye 800CW (0.1 nM total biotin-transferrin concentration) for 1 hour at 4°C. Excess biotin-transferrin was washed off with cold PBS and cells were fixed with 4% paraformaldehyde. Samples were stored at 4°C until ready to image.

### Single molecule fluorescence Imaging

The microscope used for single molecule imaging has been described elsewhere [29] and incorporated the supercontinuum light source and a zT473/543/633/700/780 filter set (Chroma). Fluorescence emission was split between two iXon^+^ cooled EMCCDs (Andor) using a TuCam (Andor) fitted with a 770dcxr dichroic beamsplitter (Chroma). HQ745/45m and HQ780LP emission filters (Chroma) were placed in the paths used to image Alexa 700/IRDye700DX and Alexa 790/IRDye800CW respectively. An HQ590/60M emission filter was used with 545 nm excitation.

Image series of 150 frames of immobilised dyes were acquired at 4 Hz under TIRF illumination without anti-fade reagents. The total power emerging from the objective was set to 2 mW for either 695 nm or 780 nm broadband illumination as appropriate. A minimum of five areas of each sample were imaged.

Image series of MCF-7 cells either unlabelled, or labeled with both transferrin-IRDye 700DX and –IRDye 800CW, were acquired at 10 Hz using epi or TIRF illumination. The power exiting the objective was the same as with the immobilised dye samples. 1.5 mW of 545 nm broadband light was also used to illuminate unlabelled MCF-7 cells. Images were acquired from unlabelled cells by illuminating the sample with each wavelength sequentially, whereas images of labeled cells were acquired whilst simultaneously illuminated with 695 nm and 780 nm light.

### Analysis of single molecule datasets

The detection and tracking of single molecules contained within multichannel image series was achieved by software that has been described in detail elsewhere [33].

Feature intensities of single molecules tracked within image series were combined to produce feature intensity histograms for each dye. Photobleaching curves were produced by plotting the sum of all feature intensities in each frame as a function of time. Histograms of time spent continuously emitting were constructed using only intensity traces that showed single step photobleaching and feature intensities consistent with the trace originating from a solitary dye molecule. The mean duty cycle of each NIR dye was calculated from the same traces of individual molecules. Fitting of Gaussian sum or single exponential functions to the data was performed using the non-linear fit function of Origin 8. Fits were considered acceptable when the adjusted-*R* ^2^ value was >0.95.

The number of detected photons, *N_P_*, was calculated from the measured fluorescence intensities, *I*, using 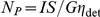, where the sensitivity of the detector, *S*, in electrons per digital level and the efficiency of the detector, *η*_det_, were taken from performance test data supplied by the manufacturer and specific to the EMCCD used. *G* was the EM gain setting used during image acquisition.

## Supporting Information

Figure S1 **Absorption and emission spectra of selected near infra-red dyes.** **A.** Absorption (blue dotted lines) and emission (red solid lines) spectra of the dyes in Table 1. **B.** Two broad excitation bands from a supercontinuum source, centred at 695 nm and 780 nm, were formed by selecting multiple channels of an acoustic optical tunable filter.
(TIF)

Figure S2 **Feature intensity histograms of selected near infra-red dyes.** Distribution of feature intensities (bin size = 100 arbitrary intensity units) constructed from single molecule intensity traces obtained from image series acquired at 4 Hz of immobilised dyes. **A.** Alexa 700, **B.** Alexa 790, **C.** IRDye 700DX and **D.** IRDye 800CW. Black lines indicate the best fit of a sum of Gaussians to the distributions.
(TIF)

Figure S3 **Photobleaching curves of selected near infra-red dyes.** Total feature intensity of immobilised dyes as a function of time, taken from image series acquired at 4 Hz. **A.** Alexa 700, **B.** Alexa 790, **C.** IRDye 700DX and **D.** IRDye 800CW. Red lines indicate the best fit of a single exponential function to the data.
(TIF)

Figure S4 **Distribution of continuous emission periods of selected near infra-red dyes.** Histograms of the periods of continuous emission contained within intensity traces obtained from image series acquired at 4 Hz of immobilised dyes. **A.** Alexa 700, **B.** Alexa 790, **C.** IRDye 700DX and **D.** IRDye 800CW. Black lines indicate the best fit of a of a single exponential function to the distributions.
(TIF)
