# Supplementary figures and images for: Multicolour Single Molecule Imaging in Cells with Near Infra-Red Dyes
Source: PLoS One. 2012 Apr 25;7(4):e36265. doi: 10.1371/journal.pone.0036265 (PMC3338497; doi:10.1371/journal.pone.0036265)

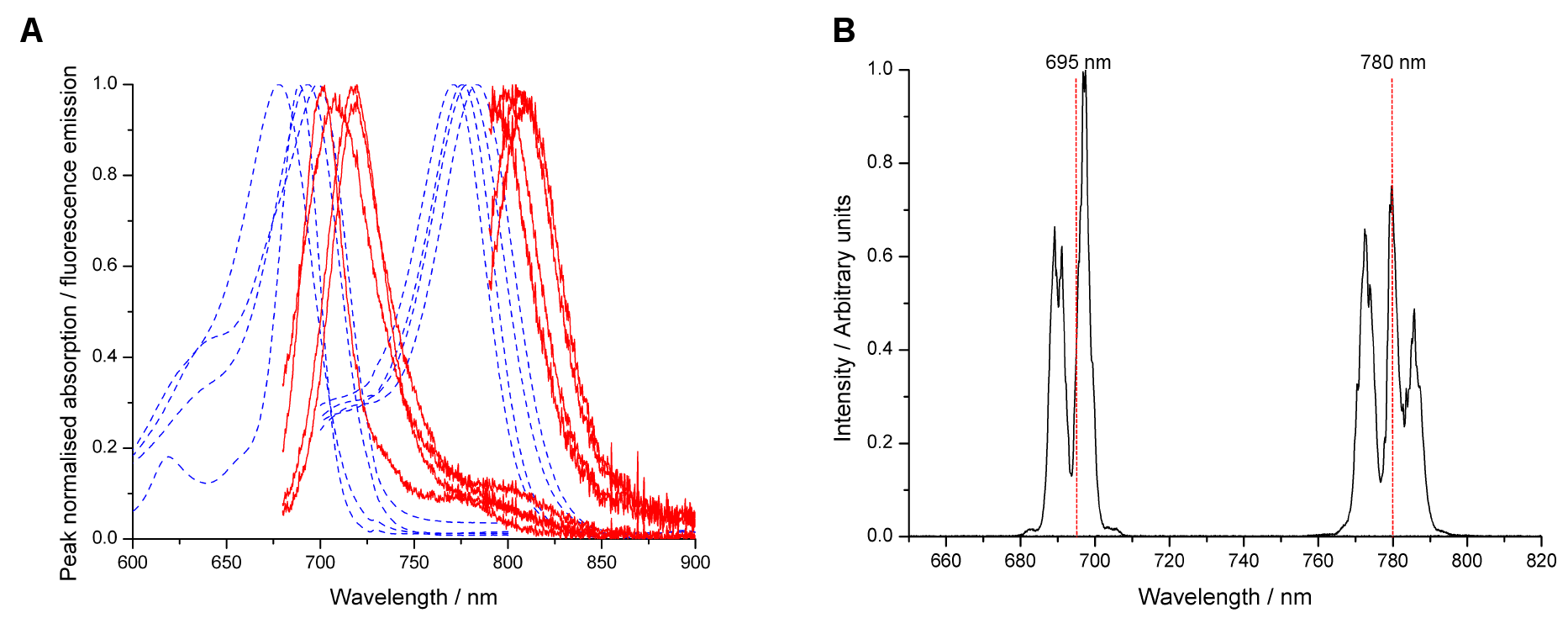

Supplement: Figure S1 — Absorption and emission spectra of selected near infra-red dyes. A. Absorption (blue dotted lines) and emission (red solid lines) spectra of the dyes in Table 1. B. Two broad excitation bands from a supercontinuum source, centred at 695 nm and 780 nm, were formed by selecting multiple channels of an acoustic optical tunable filter. (TIF) [file pone.0036265.s001.tif]

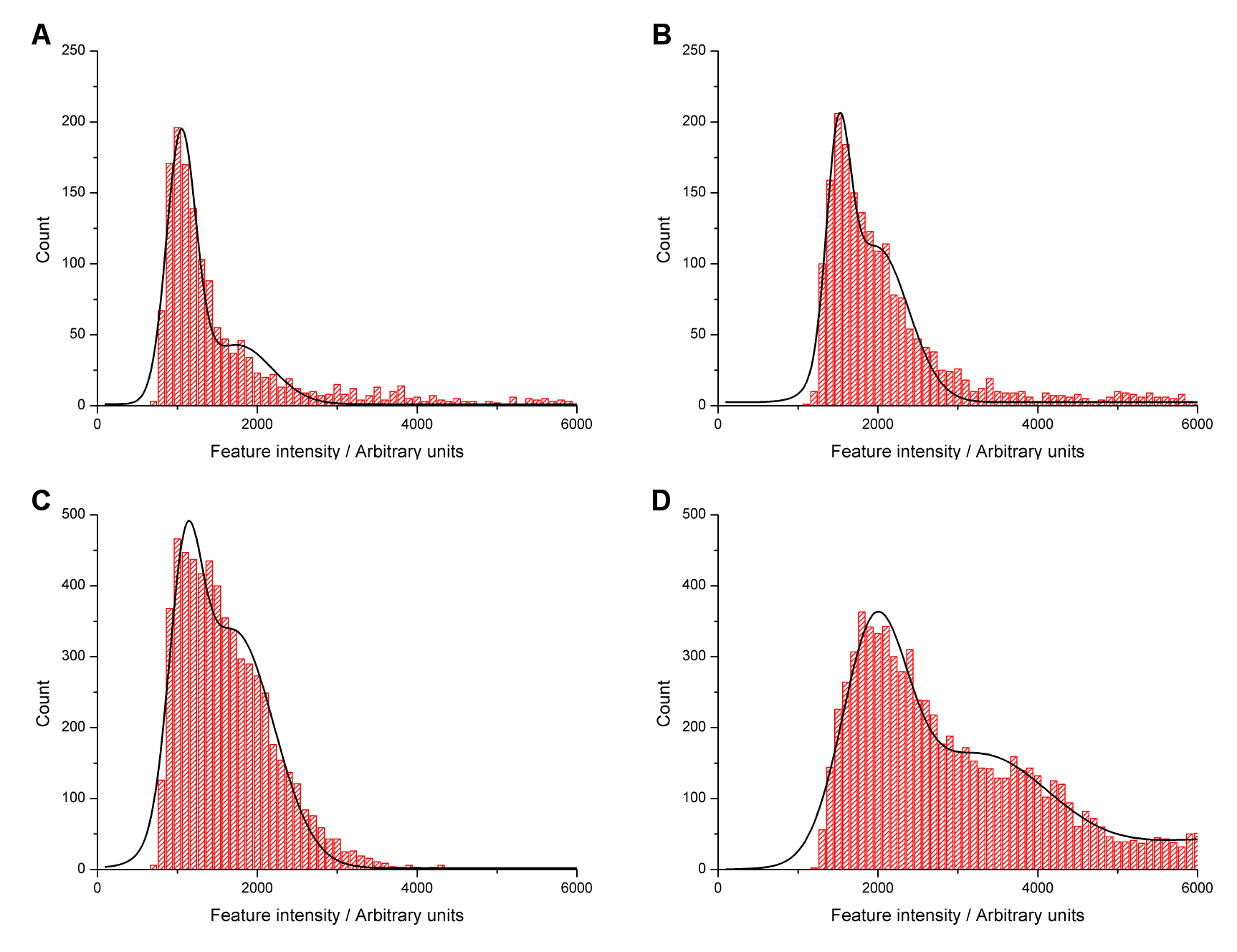

Supplement: Figure S2 — Feature intensity histograms of selected near infra-red dyes. Distribution of feature intensities (bin size = 100 arbitrary intensity units) constructed from single molecule intensity traces obtained from image series acquired at 4 Hz of immobilised dyes. A. Alexa 700, B. Alexa 790, C. IRDye 700DX and D. IRDye 800CW. Black lines indicate the best fit of a sum of Gaussians to the distributions. (TIF) [file pone.0036265.s002.tif]

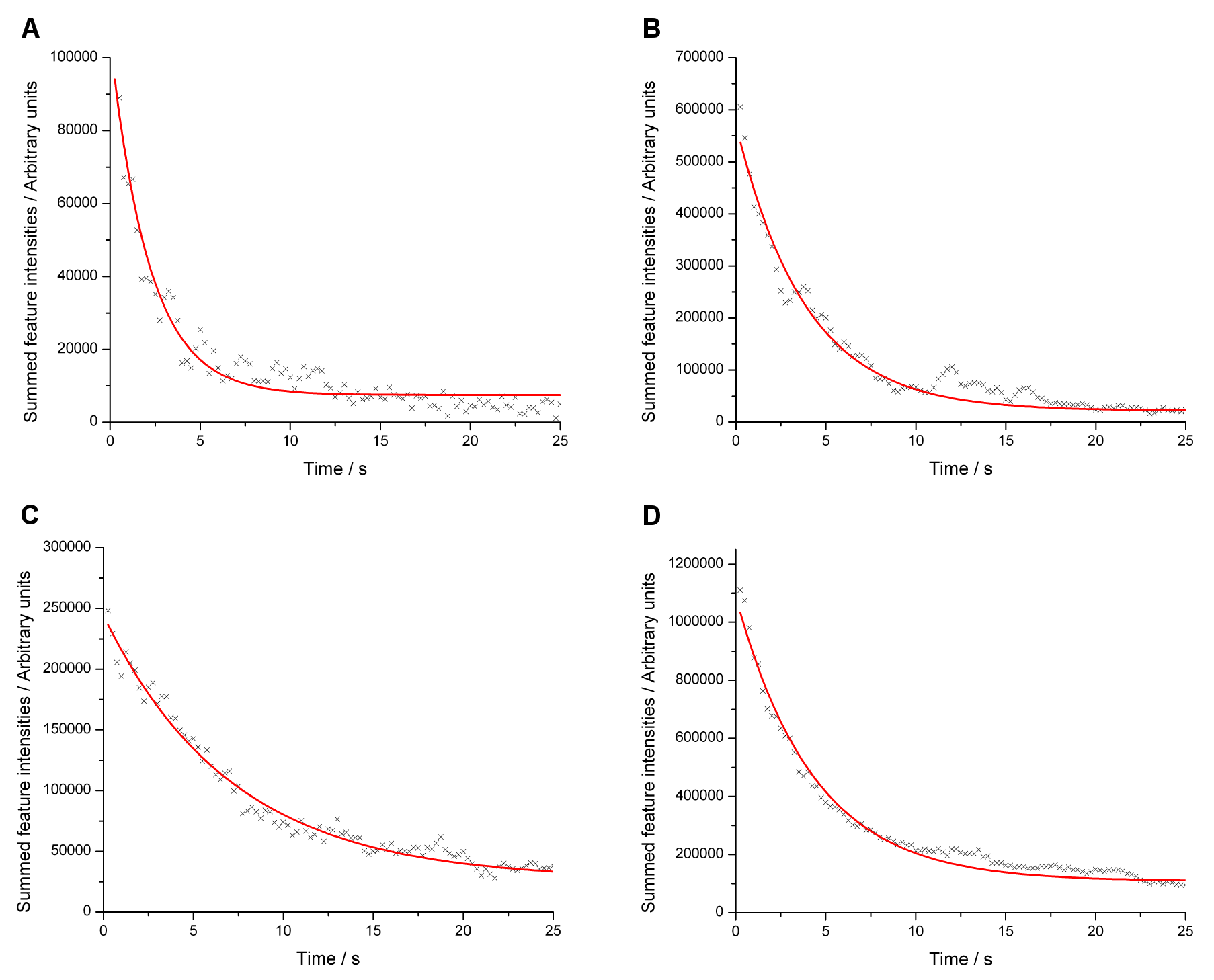

Supplement: Figure S3 — Photobleaching curves of selected near infra-red dyes. Total feature intensity of immobilised dyes as a function of time, taken from image series acquired at 4 Hz. A. Alexa 700, B. Alexa 790, C. IRDye 700DX and D. IRDye 800CW. Red lines indicate the best fit of a single exponential function to the data. (TIF) [file pone.0036265.s003.tif]

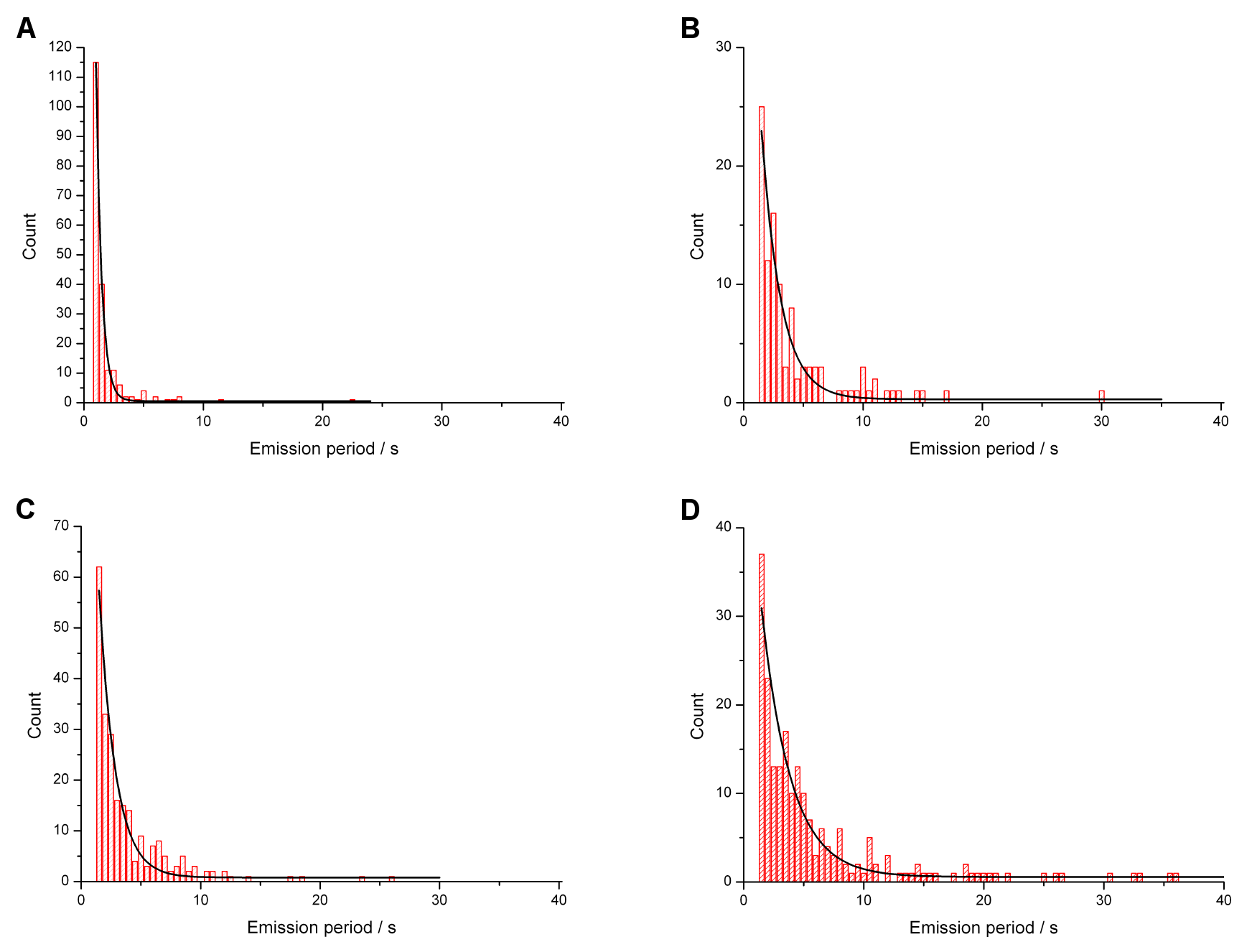

Supplement: Figure S4 — Distribution of continuous emission periods of selected near infra-red dyes. Histograms of the periods of continuous emission contained within intensity traces obtained from image series acquired at 4 Hz of immobilised dyes. A. Alexa 700, B. Alexa 790, C. IRDye 700DX and D. IRDye 800CW. Black lines indicate the best fit of a of a single exponential function to the distributions. (TIF) [file pone.0036265.s004.tif]
